# Variant analysis and biochemical investigation in two siblings with late onset idiopathic secondary erythrocytosis: A case report

**DOI:** 10.1097/MD.0000000000046424

**Published:** 2025-12-19

**Authors:** Vincenza Ylenia Cusenza, Beatrice Melli, Chiara Marraccini, Agnese Razzoli, Margherita Genitoni, Laura Albertazzi, Nesrine Gamal, Gaia Gavioli, Davide Nicoli, Enrico Farnetti, Roberto Baricchi, Erminia Di Bartolomeo, Lucia Merolle, Davide Schiroli

**Affiliations:** a Molecular Pathology, Azienda USL-IRCCS di Reggio Emilia, Reggio Emilia, Emilia-Romagna, Italy; b Obstetrics, Gynecology and Molecular Pathology Unit, Azienda USL-IRCCS di Reggio Emilia, Reggio Emilia, Emilia-Romagna, Italy; c Transfusion Medicine Unit, Azienda USL-IRCCS di Reggio Emilia, Reggio Emilia, Emilia-Romagna, Italy; d Clinical and Experimental Medicine PhD Program, University of Modena and Reggio Emilia, Modena, Emilia-Romagna, Italy; e Laboratory of Clinical Chemistry, Azienda USL- IRCCS di Reggio Emilia, Reggio Emilia, Emilia-Romagna, Italy.

**Keywords:** haemolysis, late onset, PIGV, secondary erythrocytosis, variant analysis

## Abstract

**Rationale:**

The potential etiological factors for acquired secondary erythrocytosis (SE) include sleep apnea, smoking, and renal cysts. However, there is limited evidence to consistently support an association between these factors and SE. Additionally, identifying the genetic variants underlying SE requires specific and expensive testing methods. These diagnostic challenges mean that many cases of SE are classified as idiopathic, which complicates the development of tailored diagnostic and therapeutic strategies.

**Patient concerns:**

This study examined 2 brothers (brother I [BI] and brother II [BII]) with idiopathic SE who were undergoing monthly phlebotomy.

**Diagnoses:**

A diagnosis of polycythemia vera or other acquired causes, such as pulmonary disease or malignancy, was excluded. Both subjects exhibited mild to moderate sleep apnea, while their erythropoietin levels were within the normal range.

**Interventions:**

To identify potential disease-causing variants shared by the brothers, gene panel exome-sequencing and further biochemical investigations were conducted.

**Outcomes:**

The following was identified in BI and BII: potential causative mutations in the EPAS1 gene, which were ruled out as causative factors through variant annotation and gene expression analysis; a heterozygous missense variant in the PIGV gene (p.Ala341Glu), which is known to damage proteins. The red blood cells of the brothers exhibited reduced fragility and lower hemolysis levels compared to healthy controls, with a slight increase in CD59 surface exposure.

**Lessons:**

These findings suggest that red blood cells from BI and BII are more resistant to hemolysis. However, given that PIGV is involved in glycosylphosphatidylinositol biosynthesis and that CD59 exposure affects hemolysis, further investigation is required to elucidate these pathogenic mechanisms. Molecular and biochemical characterization of patients with idiopathic SE may pave the way for identifying novel mechanisms involved in the disease.

## 1. Introduction

Erythrocytosis (also known as polycythemia) is a pathological condition involving an excessive increase in red blood cells (RBCs) mass above the normal range. It is typically diagnosed based on elevated hemoglobin and hematocrit levels.^[1,2]^ There are 2 types of erythrocytosis: primary and secondary. Primary erythrocytosis is mainly caused by an acquired mutation in the JAK2 gene within erythroid progenitor cells, leading to stem cell over-proliferation (polycythemia vera), or by congenital mutations in the EpoR and SH2B3 genes. Secondary erythrocytosis (SE), on the other hand, encompasses various disorders in which increased RBCs levels may be a consequence of hypoxia (appropriate) or altered erythropoietin production (inappropriate). Both conditions can be acquired or congenital, and are often left undiagnosed. The association with acquired causes remains elusive, although sleep apnea, smoking and renal cysts have been proposed as risk factors.^[1]^ Hereditary SE is also difficult to diagnose due to the presence of several rare variants that require specific, costly diagnostic approaches which are often inaccessible. Some studies have identified genes involved in the development of hereditary SE, but these findings are limited to a small number of cases, which are often categorized as early-onset.^[2]^ Inherited SE-causing variants have been identified in genes that play a key role in RBCs homeostasis (BPMG), hypoxia (VHL, EGLN1, PHD2 and the HIF oxygen-sensing pathway), erytrhopoyethin (EPO) production, high-oxygen affinity hemoglobin (HBB, HBA1 and HBA2) and congenital methemoglobinemia.^[1,2]^ More recently, a mutation in the PIEZO1 gene, which is associated with hemolysis, splenomegaly and iron overload, was identified in 4% of SE patients.^[3]^ However, the relationship between SE and other mutations in the SLC30A10,^[4,5]^ HFE^[6]^ and ACO1^[7]^ genes still need to be confirmed.

Furthermore, the age of onset and inheritance mechanisms vary significantly, which has a major impact on clinical evaluation.^[1]^ For this reason, most cases of SE are classified as idiopathic, hindering the development of novel, tailored diagnostic and therapeutic approaches.^[1]^ Patients regularly undergo clinical examinations and phlebotomies, which places a significant burden on the health system in terms of time and cost. Moreover, there is an ongoing debate as to whether SE may also cause or predispose individuals to more severe complications, including thrombotic events^[8,9]^ and myeloma.^[10–12]^

Using gene panel exome sequencing, we investigated the presence of disease-related gene variants in 2 siblings referred to as brother I (BI) and brother II (BII). We then conducted gene and protein expression analysis, as well as RBCs characterization, to validate the proposed variants and mechanisms.

## 2. Case presentation

### 2.1. Description of the case and clinical evaluation

The 2 brothers recruited for this study have undergone monthly phlebotomy treatment at our Transfusion Medicine Unit since receiving their initial SE diagnosis. Polycythemia vera and other acquired causes (such as EPO-inducing tumors, cardiopulmonary diseases, other malignancies, and drug treatments) were ruled out at the time of diagnosis. According to currently accepted normal laboratory ranges, the siblings exhibited elevated hematocrit levels at diagnosis (55-56%), which were maintained below 53% through periodic phlebotomy (see Table 1). Meanwhile, their EPO levels remained within the normal range (see Table 1). The patients were diagnosed with SE when they were over 50 years old (late onset). Similarly, ferritin and creatinine levels were within the normal range for both probands. Further clinical investigations revealed obstructive sleep apnea (OSA), which was classified as mild in BI and moderate in BII (Table 1). Benign cysts were found on BII’s left kidney, and neither patient reported any thrombotic events. However, analysis of their family medical history revealed that their mother had suffered from severe thrombotic events.

**Table 1:** Clinical parameters of the 2 siblings with SE (BI and BII).

	BI	BII	Range (males)
Age	72	66	
Red Blood Cells (RBC, 10^6^/µL)	5.7 ± 0.20	5.4 ± 0.18	4.3–5.9
Platelets (PLT, ×1000/µL)	170.6 ± 16	188.4 ± 13	150–450
Hemoglobin (Hb, g/dL)	17.5 ± 0.71	16.8 ± 0.70	13–17
Hematocrit (HCT, %)	52.7 ± 2.22	50.7 ± 1.77	41–50
Mean Corpuscolar Volume (MCV, fL)	93.03 ± 1.86	93.27 ± 1.90	80–100
Creatinine (mg/dL)	1.16 ± 0.05	1.10 ± 0.09	0.7–1.3
Phlebotomy	1/mo	1/mo	
Erythropoietin (EPO, U/L)	5	12	1–18.5
Ferritin (ng/mL)	151	228	40–300
Obstructive sleeping apnoea (OSA)-apnoea hypopnea index (AHI)	11/h	20/h	Normal < 5/h Mild 5–14.9/h Moderate; 15–29.9/h Severe ≥ 30/h

RBC, PLT, Hb, HCT, MCV and creatinine are mean values of the parameters collected during routine controls after the phlebotomy treatments began, between 2019 and 2024, and are expressed as mean ± SD.

BI = brother I, BII = brother II, RBCs = red blood cells, SE = secondary erythrocytosis.

While the correlation between SE and OSA remains uncertain,^[13,14]^ increased levels of EPO are generally considered to be the driving mechanism.^[15]^ As both brothers’ EPO levels were close to normal, it is unlikely that OSA alone caused their disease. Furthermore, the similar age at which BI and BII experienced the onset of the pathology and the occurrence of thrombotic events in their mother suggest the existence of an inherited pathological pattern. Consequently, BI and BII were referred from the Transfusion Medicine Unit to the Genetic Unit.^[16]^

Their exome was investigated using gene panel exome sequencing, which covers most of the genes previously associated with SE. These genes include EPAS1, VHL, EPOR, BPGM, PHD2, HBB, HBA1, HBA2, HFE, ACO1, SLC30A10 and EPO. As PIEZO1 and EGLN1 were not included in the panel, these were screened separately using Sanger sequencing with primers spanning exome sequences known to contain published SE mutations.^[3,17]^

Informed consent was obtained from probands and healthy controls in accordance with the Good Clinical Practice guidelines (DL 06/11/2007) and the European General Data Protection Regulation (EU GDPR 2016/679). The study protocol was approved by the competent ethics committee of Area Vasta Emilia Nord, Italy (protocol number: CODE:98/2015/TESS/AUSLRE).

### 2.2. Exome sequencing analysis of a selected panel of genes and variants classification

Potential disease-causing mutations and deletions were identified through exome analysis. Variants were filtered based on their presence in both siblings and a frequency of <2%.^[18]^ Genes known to be involved in SE were screened for variants with a frequency of >2% and <10%. The results of the sequencing, and subsequent variant classification and selection are summarized in Table 2. Details of gene variants with an allele frequency <1% and >1% are reported in Tables S1 and S2 (Supplemental Digital Content, https://links.lww.com/MD/Q945), respectively. For clinical exome sequencing, library preparation was performed using Illumina DNA Prep with enrichment. The sequencing chemistry integrated the DNA extraction, fragmentation, library preparation and library normalization steps, according to the manufacturer’s protocol (Illumina DNA Prep chemistry, available at https://www.illumina.com/products/by-type/clinical-research-products/trusight-one.html#tabs-105bd67c3b-item-545e4e4af2-documentation). The libraries were sequenced using a MiSeq instrument (Illumina, San Diego) using the 600-cycle (2 × 150 paired ends) MiSeq v3 Reagent Kit v3 (Illumina, San Diego). The result was ≥20× coverage on 95% of the target regions in the panel, calculated by averaging the mean coverage for each exon. The BaseSpace pipeline and Illumina Variant Studio V.3.0 were used for the variant calling and annotation in the analysis. The data were aligned to the GRCh37/hg19 human reference genome.

**Table 2:** Variants identified in both brothers (BI and BII) from the whole exome sequencing analysis.

GENE ID	Single Nucleotide Polymorphism (SNP)	Freq. % (EU)		Sift	PolyPhen	Variant type	Note/Publications
<2% frequency							
PIGV	rs139073416	.016	Het	Delet.	Probably damag.	Missense variant p.Ala341Glu	Hyperphosphatasia mental retardation syndrome homozygous or compound heterozygous^[19]^
SCNN1G	rs5738	.931	Het	Tolerat.	Benign	Missense variant p.Glu197Lys	Probably pathogenic in Bronchiectasis^[20]^
MINPP1	rs768977489	.006	Het			Downstream gene variant	In the miR4678 sequence
>2% frequency in genes involved in SE							
EPAS1	rs75591953	5.95	Hom			Intron variant	Associations of high altitude polycythemia with polymorphisms^[21]^
EPAS1	rs75984373	6.63	Hom			Intron variant	
EPAS1	rs35606117	3.365	Het			Synonymous variant	non pathogenic polymorphisms heterozygous in a patient with Idiopathic Erythrocytosis^[22]^

BI = brother I, BII = brother II.

Variants were annotated according to the Human Genome Variation Society. Their significance was assessed based on data available in scientific literature and in the ClinVar,^[23]^ Franklin Genoox^[24]^ and COSMIC^[25]^ databases, as well as SIFT and PolyPhen prediction.^[26]^ The potential target of a selected miRNA was investigated using the Gene Set Analysis database (available at https://www.gsea-msigdb.org/gsea/msigdb/human/geneset/MIR4678).

Known variants for SE in PIEZO1 and EGLN1 were investigated using Sanger sequencing. None of the known mutations found in the exome were present in BI/BII. Only exons known to contain previously known mutations were investigated for these genes. However, we cannot exclude the presence of mutations in other exons. DNA for sequencing analysis was extracted and processed as described in the Supplementary Materials (Supplemental Digital Content, https://links.lww.com/MD/Q945). PCR amplification of the DNA was performed using primer pairs specific for the exons reported in Table 1. DNA for sequencing analysis was extracted from a peripheral blood sample using the Maxwell^®^ 16 LEV Blood DNA kit (Maxwell® 16 LEV Blood DNA Kit) on the Maxwell® RSC instrument (Promega Corporation, Madison), according to the manufacturer’s specifications. DNA amplification by PCR was performed using primer pairs specific for the exons reported in Table 1. Direct sequencing of the purified PCR products was performed using BigDye Terminator v3.1 (Roche, Italy), after which the products were purified and run on an 8-capillary ABI Prism® 3500DX Genetic Analyser (Applied Biosystems, Waltham). The sequencing results were analyzed using the “Sequencher” software (Genecodes).

### 2.3. Analysis of the identified variants and biochemical characterization

Several variants in the EPAS1 (HIF2α) gene have been found to be involved in the pathogenesis of SE.^[27]^ Both probands carry a heterozygous mutation in EPAS1 (rs35606117), which was previously identified in an individual with SE from Brazil.^[22]^ However, a direct correlation with the disease remains unproven and is considered unlikely, given that it is a synonymous variant with a relatively high frequency in the general population. Additionally, both subjects have 2 other intronic mutations within a GC-rich sequence at the boundary with exon 3 (rs75591953 and rs75984373). Alterations at the intron-exon junction may cause alternative splicing and consequently alter protein expression. These variants are also known to have a higher allele frequency in Tibetan populations with altitude-related polycythemia.^[21]^ While both mutations are quite common in the European population (being present in 4–6% of individuals), the occurrence of thrombotic events in the mother indicates that recessive inheritance cannot be excluded.

**Figure 1.**
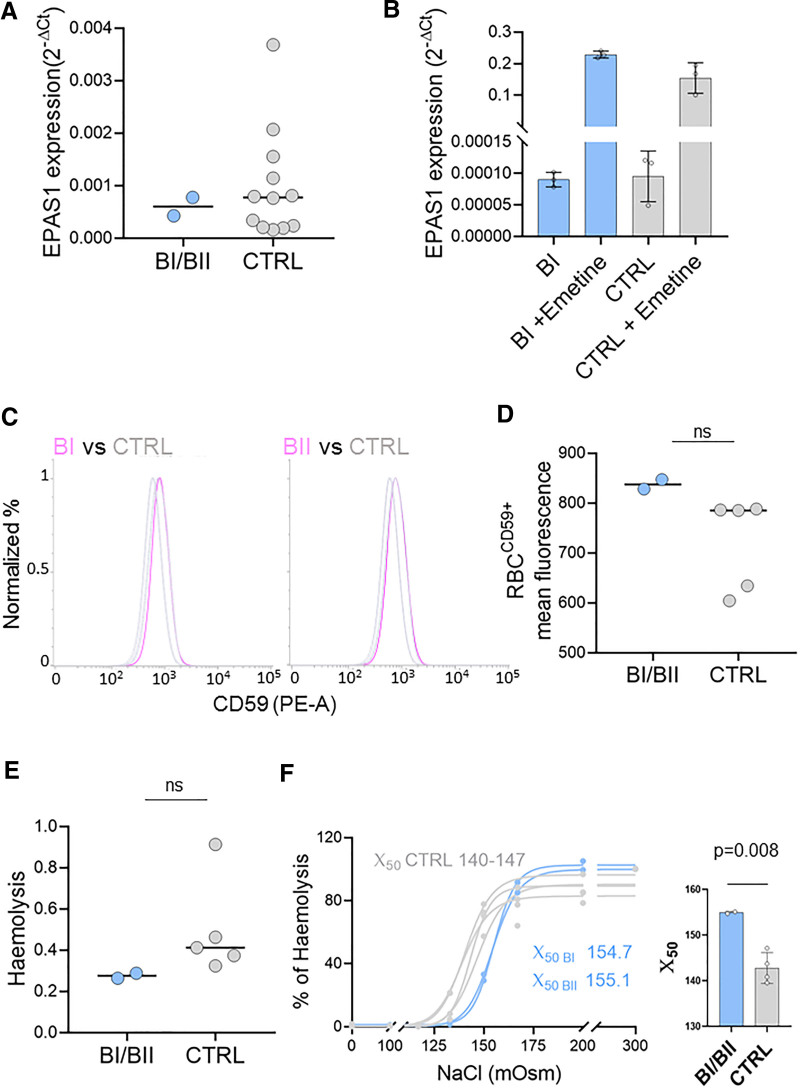
(A) EPAS1 expression in buffy coats of BI and BII (light blue) and of healthy controls, blood donors (CTRL, gray). Data are expressed as 2-ΔCt, where GAPDH and β-actin were used as housekeeping genes. (B) EPAS1 expression in blood lymphocytes isolated in vitro treated and not treated with emetine of BI (light blue) and of a healthy control, blood donor (gray), n = 3 replicates were tested for each sample. Data are expressed as 2-ΔCt, where GAPDH and β-actin were used as housekeeping genes. (C) Flow cytometry analysis of CD59 exposure on RBCs from healthy controls (gray), n = 5, and BI/BII (purple). (D) RBCs CD59^+^ mean fluorescence values of the peaks represented in C of BI/BII (light blue) and healthy subjects (CTRL, gray). (E) Haemolysis of RBCs from BI/BII (light blue) and healthy subjects (CTRL, gray). (F) In the left panel % of haemolysis of RBCs from BI/BII (light blue) and healthy subjects (CTRL, gray), the exact estimated erythrocytes mean fragility (X_50_) was indicated in the figure for BI and BII while the range values were indicated for the healthy controls (CTRL). In the right panel bars represented the X_50_ ± SD. BI = brother I, BII = brother II, RBCs = red blood cells.

To investigate the possible involvement of the EPAS1 intronic variants (rs75591953 and rs75984373) in the pathogenesis of SE, we first assessed the expression levels of the EPAS1 gene on RNA extracted from the peripheral blood of BI and BII. These were then compared with the expression levels of 5 healthy male blood donors of a similar age (60–65 years old). The EPAS1 expression levels of the 2 siblings fell within the range observed in the healthy controls, with no significant differences between the 2 brothers and the control group (Fig. 1A). As rs75591953 and rs75984373 are intronic variants, we also evaluated the production of nonfunctional transcripts resulting from alternative splicing and nonsense mediated decay (NMD). To this end, we treated peripheral blood mononuclear cells (PBMCs) from BI and a healthy control with the NMD inhibitor Emetine (100 μg/mL) for 72 hours. We then extracted mRNA from the treated and untreated samples for qPCR analysis, using an EPAS forward primer on exon 1 and an intronic primer on intron 2 (where the 2 mutations are located, Table 3). As expected, Emetine treatment led to a significant increase in EPAS1 expression; however, no difference was observed between BI and the healthy control (Fig. 1B). Overall, these results proved that the EPAS1 variants found in BI and BII did not affect EPAS1 expression or splicing, as had previously been hypothesized.^[21]^

**Table 3:** Primers used in the gene analysis.

EGLN1 primers for variant (NM_022051.3)		Sequence
c.1121A > G (p.His374Arg) rs119476045 AND c.1112G > A (p.Arg371His) rs119476044	F	TCTTTTTTTCCCCCTAAATTTAATC
	R	TCAGATGACTGTGCAACATAAATCT
c.1010dup (p.Val338fs) rs2102898170	F	AATCAGAGATCTGATTTATTAGCTG
	R	TGCACAACACTACGTTGTTAACTAC
c.950C > G (p.Pro317Arg) rs80358193	F	ATCAGTAGCCAAAAATCAGAGATCT
	R	GAGGAAAATTTTTAACTGTGTCTGT
c.461C > A (p.Ser154Ter) rs1018129986	F	GCCAAGGGAAAAGTAAAGGC
	R	TGTACTCGAGCGCCAGCTTC
PIEZO1 primers for variant (NM_001142864.4)		
Ex 25 (p.Val1223Ile) rs185326407	F	ATACCTGTTCTGGCTGGTGC
	R	GAGGCTCACCGACAGCATG
Ex 42 (p.Ala2020Thr) rs587776989	F	CACAGACATCACGTCCTCCC
	R	AGGACCCCATCAGAAACAGC
Ex 44 (p.Arg2110Trp) rs776531529 (p.Thr2127Met) rs587776991	F	CATGAGGGAGCCCACCTGA
	R	CATATCCCTGGCCCTTGTCC
Ex 51 (p.Ile2462Met)	F	CTGTACGTGTCCATCGTGCT
	R	GAGCGGTAGAGGAAGATGAGC
EPAS1 primers for gene expression		
EPAS1 Exon 1	F	CAGACCCGAAAAGAGGACGG
EPAS1 intron 2	R	AGGAACATGCTGGTAAGGCT
EPAS Exon 3	F	AAAACGAGTCCGAAGCCGAA
EPAS Exon 4	R	ACGAATCTCCTCATGGTCGC
Housekeeping		
GAPDH	F	GGTGGTCTCCTCTGACTTCAACA
	R	GTTGCTGTAGCCAAATTGTTGT
β-Actin	F	ACCTTCTACAATGAGCTGCG
	R	CCTGGATAGCAACGTACATGG

F = forward, R = reverse.

To analyze EPAS1 expression, RNA was extracted from the buffy coat of whole blood samples, collected in 3 mL EDTA tubes (Vacutainer), using Maxwell® 16 LEV simplyRNA Blood kit with the RNA isolation protocol (ref. AS1310; Promega Corporation). RNA from PBMCs was extracted using a NEB Monarch Total RNA miniprep kit. Reverse transcription (RT) was performed using the PrimeScript™ RT Master Mix kit (Takara Bio, Inc., Kusatsu, Shiga, Japan) according to the manufacturer’s protocol. Gene expression was performed via qPCR using GoTaq qPCR Master Mix (Promega) and the 2^−∆Ct^ method of quantification, following the manufacturer’s protocols. β-Actin and GAPDH were used as the reference genes in RT-qPCR. The primer conditions are reported in Table 3.

PBMCs were isolated from BI and a healthy control using the standard protocol for Ficoll™ (Sigma-Aldrich) density gradient separation, and cultured on 12-well plates (EuroClone, Italy) in RPMI medium (VWR) with 10% FBS (EuroClone, Italy) at the final concentration of 1 × 10^6^ cells at 37°C, 5% CO_2_. NMD was tested by treating the PBMCs with 100 μg/mL of the NMD inhibitor Emetine^[28]^ (E2375, Sigma-Aldrich) for up to 72 hours. mRNA was extracted from the treated and untreated PBMCs and tested with qPCR (as described before) with an EPAS forward primer on exon 1 and an intronic primer on intron 2 (where the 2 mutations are located, Table 3).

We also investigated whether other variants identified in both subjects (BI and BII) with an allele frequency of <1% (Table S1, Supplemental Digital Content, https://links.lww.com/MD/Q945) might cause erythrocytosis directly or indirectly via hypoxia. We identified a downstream variant in a sequence coding for the microRNA miR4678 in the MINPP1 gene. To our knowledge, this miRNA has not previously been associated with any pathology. As we did not find any genes known to be involved in SE among the possible target genes of miR4678 (https://www.gsea-msigdb.org/gsea/msigdb/human/geneset/MIR4678), further functional analysis would be necessary to elucidate this aspect. The missense variant p.Glu197Lys of SCNN1G, which encodes for the amiloride-sensitive epithelial Na^+^-channel ENaCγ, was previously identified in 2 patients with severe bronchiectasis.^[20]^ Defective SCNN1G variants are known to lead to impaired alveolar and lung function,^[29]^ which could hypothetically cause hypoxia and SE. However, in silico predictions (Polyphen/Sift) suggest that the p.Glu197Lys missense variant is not pathogenic, and no functional assay has confirmed its association with the disease. Furthermore, the 2 siblings had no obvious respiratory disease other than mild OSA, and their EPO levels were normal.

The PIGV rs139073416 variant (present in both BI and BII) has a low allele frequency in the European population (0.016%), and the p.Ala341Glu mutation is classified as deleterious and potentially damaging by Sift/Polyphen prediction. Furthermore, homozygous or compound heterozygous missense mutations of the Ala341Glu variant have been identified in patients with hyperphosphatasia mental retardation syndrome.^[19,30]^ PIGV encodes for an enzyme involved in the glycosylphosphatidylinositol (GPI)-anchor biosynthesis pathway, which has been found to be defective in a group of congenital disorders of glycosylation^[31]^ characterized by altered exposure of GPI-linked proteins on the cell surface. Since BI and BII were found to be heterozygous for this mutation, only part of their PIGV is defective, hich could lead to diminished GPI production and exposure of GPI-linked proteins. Mutations in another enzyme in the GPI synthesis pathway, PIGA, cause paroxysmal nocturnal hemoglobinuria,^[32]^ in which hematopoietic stem cells produce RBCs lacking CD55 and CD59, 2 surface proteins that prevent RBCs hemolysis.

To investigate whether BI and BII had an altered exposure of GPI-anchored proteins, we measured CD59 expression on the surface of RBCs. Interestingly, CD59 exposure was not reduced on RBCs surfaces compared to those of healthy controls, as is typically observed in paroxysmal nocturnal hemoglobinuria. However, it was slightly increased (Fig. 1C and D). The values measured for BI and BII were outside the range measured in the controls, although this did not reach statistical significance. This may be due to the small number of affected individuals analyzed in this case report. In the same set of samples, we also compared RBCs fragility and hemolysis.^[33]^ Consistent with the CD59 quantification results, we observed decreased hemolysis and erythrocytes fragility in BI and BII (Fig. 1E and F). In this case too, the difference in hemolysis was not significant, but we found a significantly lower erythrocyte fragility when comparing the X_50_ values (*P*-value .008). These findings suggest that the PIGV variant, which potentially influences the exposure of the GPI-anchored CD59 protein exposure, might confer increased resistance to hemolysis in the 2 probands. However, establishing a direct link between these findings requires further investigation.

Samples were analyzed from 1 mL of whole blood collected in blood count EDTA tubes (Vacutainer) within a few hours of collection. Flow cytometry analysis was performed using a BD FACSLyric (BD Bioscience). RBCs were gated based on their physical properties (side and forward scattering) and stained with an anti-CD59-PE monoclonal antibody (CYT-59PE7, BD Bioscience). The RBCs in the blood count sample were analyzed by dilution to a ratio of 1:50 in PBS, followed by incubation in the dark for 20 minutes with 20 μL of the CD59-PE antibody in a total volume of 100 μL. The prepared sample was then further diluted in 1 mL of PBS, centrifuged at 2000 g for 3 minutes, and the pellet was reconstituted in a final volume of 300 μL PBS prior to acquisition. RBCs fragility and hemolysis were investigated in whole blood samples collected in blood count tubes containing EDTA (Vacutainer), as previously described.^[33]^ Briefly, hemolysis of RBCs was assessed using the Harboe direct spectrophotometric method by measuring free hemoglobin (HbO_2_) absorbance at 415 nm (ε = 512 mM^−1^ cm^−1^). Hemolysis percentage was calculated based on free hemoglobin in the supernatant, total hemoglobin and hematocrit. Erythrocyte osmotic fragility was evaluated in triplicate. A total of 5 μL of RBCs were incubated with 95 μL NaCl solutions with a decreasing osmolarity (300 to 0 mOsm), and then centrifuged (2500*g*, 1 min). Hemoglobin release in the supernatant was measured at 560 nm in 96-well plates (GloMax, Promega). The data were fitted to a sigmoidal curve, and the X_50_ (the mean osmolarity causing 50% lysis) was calculated.

## 3. Discussion

In this study, we analyzed the exomes of 2 siblings with idiopathic SE, normal EPO levels, mild-to-moderate obstructive sleep apnea, and a family history of thrombotic events, which suggests a potential shared genetic predisposition. While EPAS1 (HIF2α) mutations are a well-established cause of SE, annotation and gene expression analyses ruled out the pathogenicity of the identified EPAS1 variants in both patients. Additional rare variants were identified in MINPP1 (within miR4678, allele frequency 0.006) and SCNN1G. However, the relevance of these variants to SE remains speculative.

Notably, both siblings were found to carry a heterozygous missense variant, p.Ala341Glu, in PIGV, a gene in the GPI-anchor biosynthetic pathway. Moreover, BI and BII exhibited mildly increased CD59 expression on RBCs, reduced red cell fragility, and lower hemolysis. Pathogenic variants in PIGV typically cause hyperphosphatasia mental retardation syndrome when present in homozygous or compound heterozygous states.^[19]^ Defects in GPI-anchor biosynthesis are implicated in various human diseases; in particular, mutations in PIGA^[32]^ and, as was recently discovered, PIGV^[34]^ mutations have been found to be causative of paroxysmal nocturnal hemoglobinuria.

Nevertheless, the subtle RBCs phenotype described for the 2 siblings suggests a counterintuitive effect of possible heterozygous PIGV deficiency. One possible explanation is that partial loss of PIGV function in heterozygosity leads to residual wild-type activity. This could subtly alter GPI-anchor biosynthesis and enhance the membrane presentation of CD59. This is similar to what has been observed for CD55 in PIGS-deficient or ERAD-pathway knockout models.^[35]^

A recent study elegantly demonstrated that GPI-anchor biosynthesis deficiency can lead to the accumulation of incomplete precursors, thereby enhancing overall GPI biosynthesis. In our case, this mechanism, combined with residual wild-type activity, could result in increased CD59 exposure. This could lead to prolonged RBCs survival. CD59 inhibits the formation of the complement membrane attack complex and, under normal conditions, protects erythrocytes from complement-mediated lysis. Conversely, in patients with paroxysmal nocturnal hemoglobinuria, where CD59 (and CD55) are deficient, RBCs become highly susceptible to intravascular hemolysis. However, to our knowledge, increased CD59 expression has never been shown to protect RBCs from hemolysis. Interestingly, elevated CD59 levels in various tumor types have been associated with more aggressive behavior, suggesting that cancer cells may exploit this mechanism to evade complement-mediated lysis.^[36–38]^

Unfortunately, our current knowledge of heterozygous carriers of variants in genes involved in GPI biosynthesis is limited.^[39]^ Hematological parameters in such individuals have not been systematically studied, and when reported, carriers are generally asymptomatic despite harboring pathogenic variants (e.g. in PIGG and PIGV).^[40–42]^ Nevertheless, a few observations are consistent with reports that heterozygous carriers of GPI biosynthesis gene variants often exhibit subtle biochemical changes without evident clinical manifestations. Notably, a heterozygous PIGA carrier with a non-skewed X-inactivation pattern exhibited reduced GPI-AP expression in subpopulations of blood cells despite the absence of evident clinical symptoms, highlighting that heterozygous variants may exert mosaic molecular effects.^[43]^ Moreover, heterozygous carriers (i.e., parents of affected children) with PIGU mutations showed subtle differences in GPI-AP staining when compared to healthy controls.^[44]^ In this context, the gene-dosage effect is a coherent hypothesis: the heterozygous PIGV variant may slightly modulate GPI-anchor metabolism, resulting in measurable changes in CD59 expression and RBC survival without causing severe disease. This concept aligns with our observation of modestly increased CD59 levels and reduced hemolysis, although the proposed mechanism is speculative and the direct link has yet to be established.

Our findings also suggest that a milder phenotype may only emerge in heterozygous individuals under specific circumstances, such as advanced age or the presence of additional genetic or environmental stressors, as observed in BI and BII. The slight but consistent increase in CD59 expression may also reflect alternative protective mechanisms that reduce hemolysis, which may be dependent on or independent of GPI-anchor biosynthesis. Notably, newly identified classes of GPI-anchored proteins^[45]^ have revealed previously unrecognized pathological pathways in RBCs, suggesting that other compensatory mechanisms may be at play.

Additional mechanisms of hemolysis resistance should also be considered. For example, ion homeostasis dysregulation in RBCs is known to cause hereditary xerocytosis, a disorder that is occasionally associated with polycythemia.^[46]^ While this mechanism can be ruled out in our cases given the normal mean corpuscular hemoglobin concentration, alterations in membrane structure or transmembrane protein function could also decrease hemolysis and extend RBC lifespan. Traditionally, SE has been attributed to increased erythropoiesis driven by hypoxia or dysregulation of the oxygen-sensing pathway. However, our observations suggest that reduced RBC turnover, with prolonged cell survival, may represent an additional or alternative mechanism contributing to SE in certain genetic contexts.

Taken together, these findings suggest that impaired RBCs clearance may play a role in the pathophysiology of SE. Further experimental validation is needed to clarify the mechanistic relationship between the heterozygous PIGV variant, the modest increase in CD59 expression, and reduced hemolysis. Nevertheless, the idea that SE may arise from both increased erythropoiesis and decreased RBCs turnover provides a new direction for research. Improving our understanding of these mechanisms could lead to better characterization, diagnosis, and treatment of SE. Future studies integrating molecular, biochemical, and in vitro approaches are essential for addressing this gap.

## Author contributions

**Conceptualization:** Chiara Marraccini, Nesrine Gamal, Roberto Baricchi, Erminia Di Bartolomeo, Lucia Merolle, Davide Schiroli.

**Data curation:** Vincenza lenia Cusenza, Beatrice Melli, Davide Nicoli, Enrico Farnetti, Davide Schiroli.

**Formal analysis:** Beatrice Melli, Agnese Razzoli, Davide Nicoli, Enrico Farnetti.

**Investigation:** Vincenza Ylenia Cusenza, Beatrice Melli, Agnese Razzoli, Margherita Genitoni, Laura Albertazzi, Gaia Gavioli, Davide Nicoli, Enrico Farnetti.

**Methodology:** Vincenza Ylenia Cusenza, Beatrice Melli, Davide Nicoli, Enrico Farnetti.

**Project administration:** Roberto Baricchi.

**Resources:** Roberto Baricchi.

**Supervision:** Nesrine Gamal, Roberto Baricchi, Davide Schiroli.

**Validation:** Vincenza Ylenia Cusenza.

**Visualization:** Davide Schiroli

**Writing – original draft:** Chiara Marraccini, Lucia Merolle, Davide Schiroli.

**Writing – review & editing:** Chiara Marraccini, Margherita Genitoni, Erminia Di Bartolomeo, Lucia Merolle, Davide Schiroli.

## Supplementary Material


